# Potent Suppression of Kv1.3 Potassium Channel and IL-2 Secretion by Diphenyl Phosphine Oxide-1 in Human T Cells

**DOI:** 10.1371/journal.pone.0064629

**Published:** 2013-05-22

**Authors:** Ning Zhao, Qian Dong, Li-Li Du, Xiao-Xing Fu, Yi-Mei Du, Yu-Hua Liao

**Affiliations:** Research Center of Ion Channelopathy, Institute of Cardiovascular Diseases, Union Hospital, Tongji Medical College, Huazhong University of Science and Technology, Wuhan, P.R. China; Temple University, United States of America

## Abstract

Diphenyl phosphine oxide-1 (DPO-1) is a potent Kv1.5 channel inhibitor that has therapeutic potential for the treatment of atrial fibrillation. Many other Kv1.5 channel blockers also potently inhibit the Kv1.3 channel, but whether DPO-1 blocks Kv1.3 channels has not been investigated. The Kv1.3 channel is highly expressed in activated T cells, which is considered a favorable target for immunomodulation. Accordingly, we hypothesized that DPO-1 may exert immunosuppressive and anti-inflammatory effects by inhibiting Kv1.3 channel activity. In this study, DPO-1 blocked Kv1.3 current in a voltage-dependent and concentration-dependent manner, with IC_50_ values of 2.58 µM in Jurkat cells and 3.11 µM in human peripheral blood T cells. DPO-1 also accelerated the inactivation rate and negatively shifted steady-state inactivation. Moreover, DPO-1 at 3 µM had no apparent effect on the Ca^2+^ activated potassium channel (K_Ca_) current in both Jurkat cells and human peripheral blood T cells. In Jurkat cells, pre-treatment with DPO-1 for 24 h decreased Kv1.3 current density, and protein expression by 48±6% and 60±9%, at 3 and 10 µM, respectively (both p<0.05). In addition, Ca^2+^ influx to Ca^2+^-depleted cells was blunted and IL-2 production was also reduced in activated Jurkat cells. IL-2 secretion was also inhibited by the Kv1.3 inhibitors margatoxin and charybdotoxin. Our results demonstrate for the first time that that DPO-1, at clinically relevant concentrations, blocks Kv1.3 channels, decreases Kv1.3 channel expression and suppresses IL-2 secretion. Therefore, DPO-1 may be a useful treatment strategy for immunologic disorders.

## Introduction

Diphenyl phosphine oxide-1 (DPO-1) represents a novel class of Kv1.5 blocker that has been documented to selectively prolong atrial action potential duration (APD) without exerting significant effects on ventricular APD [1], [2]. *In vivo*, DPO-1 has been shown to increase the atrial effective refractory period and terminate atrial arrhythmias within 60 s in dog [3], [4]. However, in cholinergic atrial fibrillation (AF) models in pig, DPO-1 at 0.5 µM did not terminate AF in a majority of experiments [5]. Recently, we have found that DPO-1 preferentially blocks the open state of the Kv1.5 channel by binding with several key residues in the S5-pore loop-S6 domains [1]. Interestingly, all of these Kv1.5 key residues are also highly conserved in the Kv1.3 channel [6], [7], which provides a molecular structural basis for why many Kv1.5 blockers (e.g., AVE0118 and S0100176) also potently inhibit the Kv1.3 channel [8], [9]. Therefore, the structural homology among these channel subtypes suggested that DPO-1 may block Kv1.3 channels as well.

Kv1.3 is the predominant voltage-gated potassium channel expressed in T cells, and shows C-type inactivation similar to other *Shaker* isoforms (Kv1). In human T cells, Kv1.3 regulates cell membrane potential and promotes sustained Ca^2+^ influx required for T-cell receptor-mediated cell activation, migration, proliferation, and IL-2 secretion [10]. Therefore, Kv1.3 channel blockers have immunosuppressive and anti-inflammatory properties [11], [12]. Moreover, autoimmune disease-related effector memory T cells (T_EM_) expressed significantly higher levels of Kv1.3 channel after activation in multiple sclerosis [13], rheumatoid arthritis [14], type-1 diabetes [15], and psoriasis [16]. Selective inhibition of Kv1.3 channels resulted in the down-regulation of T_EM_ activities, and ameliorated autoimmune diseases in animal models [16], [17], [18]. Therefore, developing Kv1.3 channel blockers could be useful treatment strategy for immunologic disorders.

To date, no studies have evaluated the ability of DPO-1 to block Kv1.3 channels. *Xenopus* oocytes or mammalian cell lines (human embryonic kidney 293 and Chinese hamster ovary cells) have been widely used to characterize the electropharmacological properties of Kv1.3 channel blockers [19], [20]. However, use of these cell types does not reflect the true physiological environment of the Kv1.3 channel in human T cells and does not allow the immunomodulatory effects of Kv1.3 channel blockers to be evaluated. In the present study, we compared the effects of DPO-1 on Kv1.3 channels in the Jurkat cell line and in human peripheral blood T cells, and further investigated the effects of DPO-1 on Ca^2+^ influx, and IL-2 secretion in Jurkat cells. In the current study we demonstrate for the first time that DPO-1 blocks Kv1.3 currents, decreases Kv1.3 channel expression, attenuates Ca^2+^ influx, and inhibits IL-2 production.

## Materials and Methods

### Ethics statement

In this study, the study protocol with human blood samples was approved by the Ethics Committee of Tongji Medical College of Huazhong University of Science and Technology. Human blood samples were taken from healthy blood donors, who were provided written informed consent for the collection of blood and subsequent T cells isolation and analysis.

### Cell preparation and culture condition

The human leukemia T-cell line, Jurkat E6-1, was obtained from the American Tissue Culture Collection (ATCC, Rockville, MD, USA). Human peripheral blood T cells were separated from whole blood samples using Ficoll gradients and purified by negative selection using CD4^+^ T Cell Isolation Kit (Miltenyi Biotec, Bergisch-Gladbach, Germany). All cells were grown in culture medium consisting of RPMI 1640 supplemented with 10% heat-inactivated FBS, 10 mM HEPES, 2 mM glutamate, 100 U/mL penicillin/streptomycin and were maintained at 37°C in a humidified 95% air and 5% CO_2_ atmosphere. Jurkat cells were stimulated with 50 ng/mL phorbol ester (PMA, Sigma-Aldrich, St Louis, MO) and 5 µg/mL phytohematogglutinin (PHA, Sigma-Aldrich). DPO-1 (Tocris Biosciences, Bristol, UK) at 3 µM or 10 µM was added at the onset of stimulation or 30 minutes prior to stimulation. Margatoxin (MgTX; 10 nM) and charybdotoxin (ChTX; 100 nM) were used as positive controls to block Kv1.3 channels (both from Alomone Laboratories Ltd, Jerusalem, Israel).

### Electrophysiological recordings

All currents were recorded using a whole-cell patch configuration at room temperature. The external Ringer solution was (in mM): 137 NaCl, 4 KCl, 1.8 CaCl_2_, 1 MgCl_2_, 10 glucose, and 10 HEPES, adjusted with NaOH to pH 7.4. For Kv1.3 current measurements, the pipette solution (in mM) consisted of 130 KCl, 1 MgCl_2_, 5 EGTA, 5 Mg-ATP, 10 HEPES, adjusted to pH 7.2 with KOH. Kv1.3 currents were elicited by depolarizing pulses ranging from −80 mV to +60 mV in 10 mV increments from the holding potential of −80 mV. The membrane potential was measured in zero current (I = 0) mode using whole-cell patch technique. K_Ca_ currents were elicited by using 200 ms voltage ramps from −120 mV to +60 mV, applied every 30 s from the holding potential of −40 mV. For K_Ca_ measurements, the pipette solution contained (in mM): 130 potassium aspartate, 10 EGTA, 8.55 CaCl_2_, 2.08 MgCl_2_, and 10 HEPES, adjusting to pH 7.2 using KOH, with a calculated free [Ca^2+^] of approximately 1 µM. For some experiments recording K_Ca_, the bath solution was replaced by a K^+^ Ringer solution, which featured identical ingredients, except that all NaCl was substituted by KCl.

The pCLAMP 9.0 and Origin 8.5 (OriginLab Corporation, Northampton, MA, USA) software were used for data acquisition and analysis. Membrane conductance (*G*) was defined as *I*/(*V*−*E*_rev_), where *I* is the peak amplitude, *V* is the test voltage, and *E_rev_* is the reversal potential (−90 mV) of Kv1.3 currents [21]. The steady-state inactivation curve was studied by using a double-pulse protocol, in which the test voltage was stepped to +40 mV, 100 ms long, and preceded by 30 s preconditioning pulses from −80 to 0 mV in 10 mV steps. The data for activation and steady-state inactivation were fitted with the Boltzmann equation to get the half-maximum voltage of activation or inactivation voltage (*V*_1/2_) and slope factor. The percent inhibition (% inhibition) of the current was defined as [(*I*_control_−*I_drug_*)/*I_control_*]*100%. The concentration required for 50% inhibition of the current (IC_50_) was determined by fitting the data with the Hill equation.

### Western blotting

Jurkat cell total lysate was prepared according to standard procedures. Cells were washed twice in cold PBS, homogenized with PMSF buffer (Roche, Basel, Switzerland), and centrifuged at 12,000 rpm for 20 min. Protein concentrations of the supernatants were measured using a BCA kit (Pierce, Rockford, IL, USA). Samples containing 40 µg of total protein were boiled in SDS loading buffer, separated on a 10% SDS-PAGE gel, and transferred to nitrocellulose membranes. The membranes were blocked in 5% dry milk, supplemented with 0.3% Tween-20 TBS, for 1 h at room temperature and probed with anti-Kv1.3 (diluted 1∶500, Abcam, MA, USA) or anti-β-actin (diluted 1∶1000, Santa Cruz Biotechnology, Inc., CA, USA) overnight at 4°C. The membranes were washed three times and incubated with anti-rabbit or anti-mouse peroxidase-conjugated IgG (diluted 1∶3000, Antigene, Wuhan, China) for 1 h at room temperature. Bands were visualized using West Pico Chemiluminescent Substrate (Thermo Scientific Inc., Bremen, Germany) and quantified by densitometric analysis using Quantity One software (Bio-Rad Laboratories, Hercules, CA).

### Single cell Ca^2+^ measurements

Jurkat cells were loaded with 2 µM fluo-3 AM (Invitrogen, Carlsbad, CA) at room temperature for 30 min and washed twice with Ca^2+^ free Ringer's solution. Cells were allowed to adhere to laminin-coated glass coverslip chambers (Sigma-Aldrich) on the stage of a Nikon (Tokyo, Japan) A1SI laser confocal microscope equipped with a 40× (Uplan/Apo, N.A. 1.0) objective and illuminated at 488 nm. Fluorescence emissions at 530 nm were captured with a charge-coupled device camera, digitized, and analyzed using the NIS-Elements microscope imaging software program. Images were recorded at intervals of 10 s. Ca^2+^ release was induced by 1 µM thapsigargin (TG, Alomone) in Ca^2+^ free Ringer solution. After 5 min, normal Ringer solution (2 mM CaCl_2_) was reintroduced. The fluorescent index ΔF/F_0_ was calculated as the average Ca^2+^ response (ΔF = F-F_0_, F_0_ is the mean value of background fluorescence).

### IL-2 secretion measurements

IL-2 production in Jurkat cells was measured using an ELISA kit (eBioscience, San Diego, CA, USA) following the manufacturer's instructions. Cells were centrifuged at 1500 rpm for 10 min, and the supernatants were collected to measure IL-2 concentrations. Reactions were performed in 96-well plates coated with the capture antibody and stopped with phosphoric acid (1 M). Absorbance was measured with an automatic plate reader at 450 nm. Each experiment was repeated at least three times in duplicate.

### Statistical analysis

Results are reported as mean±SEM. The significance of any differences before and after drug treatment was evaluated using a paired *t*-test. Comparisons between groups were carried out by analysis of variance with Turkey's post-test. Significance was set at P<0.05.

## Results

### DPO-1 blocks Kv1.3 channel currents in human T cells

**Figure 1:**
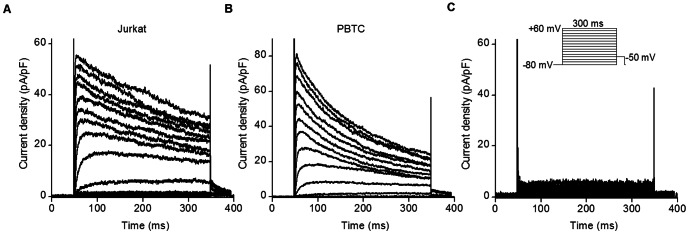
Jurkat cells and human peripheral blood T cells (PBTC) express Kv1.3 currents. Original current traces were elicited by 300 ms depolarizing pulses from a holding potential of −80 mV to test potentials between −80 mV and +60 mV, with 10 mV steps in Jurkat cells (**A**), in peripheral blood T cells (**B**) and after application of 10 nM MgTX (**C**), a Kv1.3 blocker in Jurkat cells.

We first defined the Kv channels expressed in Jurkat cells and human peripheral blood T cells. Kv currents were elicited by 300 ms pulses at test potentials ranging from −80 to +60 mV with 10 mV steps, from a holding potential of −80 mV. The outward currents at potentials positive to −40 mV were characterized by rapid activation and slow inactivation as reported previously (Figure 1*A* and 1*B*) [22], [23]. After application of 1 nM MgTX, a selective Kv1.3 channel blocker [24], the currents were nearly completely abolished (Figure 1*C*), confirming that the Kv1.3 current was the predominant component of the outward current in Jurkat cells and human peripheral blood T cells.

**Figure 2:**
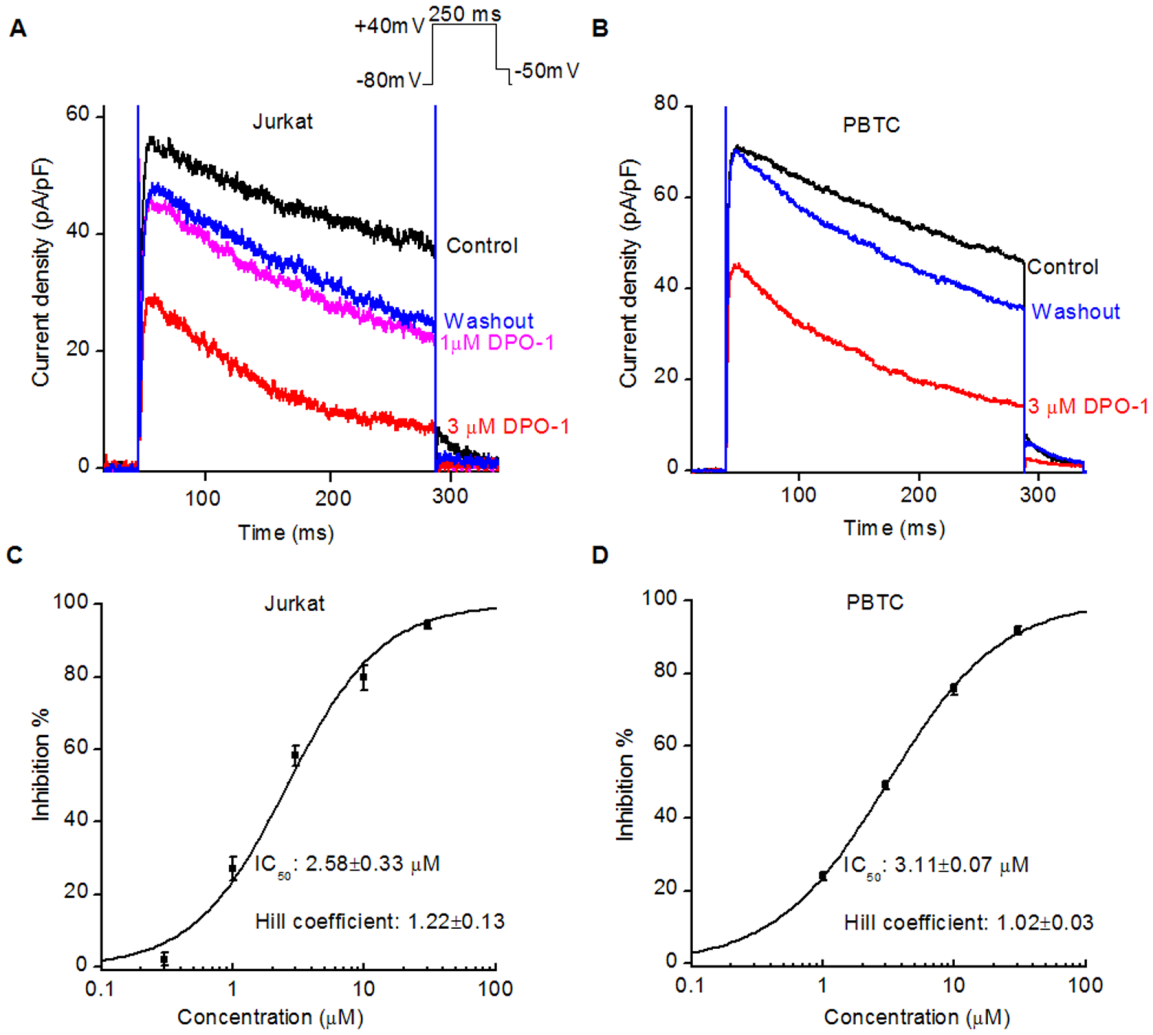
Concentration-dependent blockade of Kv1.3 channels by DPO-1 in human T cells. Currents were elicited by 250 ms depolarizing pulses from a holding potential of −80 mV to +40 mV every 30 s. Representative current traces were superimposed in Jurkat cells (**A**) and human peripheral blood T cells (**B**) before (control, in black), during perfusion with DPO-1 (pink and red curves), and after washout (in blue). Summarized data from Jurkat cells (**C**) and human peripheral blood T cells (**D**) showing the dose-response curve fitted with the Hill equation. Data were expressed as mean±SEM from 6 cells for each concentration.

We then examined the concentration-dependent inhibitory effects of DPO-1 on Kv1.3 currents in Jurkat cells and human peripheral blood T cells. Cells were perfused externally with different concentrations of DPO-1 from 0.3 to 30 µM. The peak current amplitude generated by stimulation at +40 mV was used as the index of inhibition (Figure 2*A and 2B*). A nonlinear least-square fit of the Hill equation to the concentration response data yielded an IC_50_ of 2.58±0.33 µM in Jurkat cells and 3.11±0.07 µM in peripheral blood cells. The Hill coefficient was 1.22±0.13 in Jurkat cells and 1.02±0.03 in peripheral blood T cells (Figure 2*C and 2D*). The Hill coefficient was very close to 1, which suggests that DPO-1 blocks the channel in a 1∶1 stoichiometric ratio.

**Figure 3:**
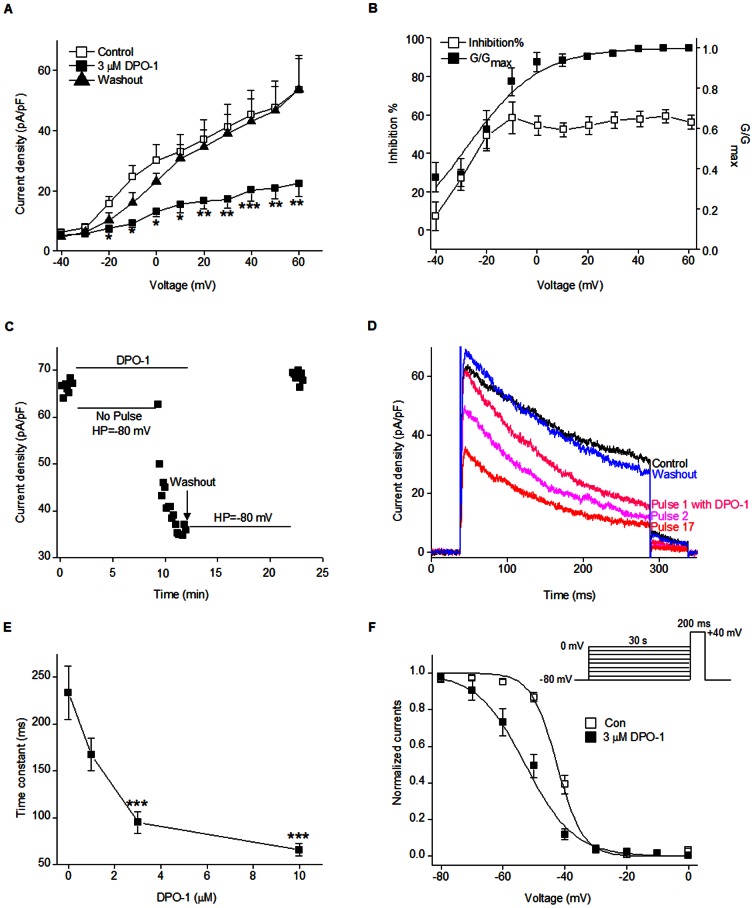
DPO-1 blocked the Kv1.3 channel in Jurkat cells. (**A**) An average current density-voltage relationship for peak Kv1.3 current in the absence (open squares), presence of 3 µM DPO-1 (filled squares), and washout (filled triangles), **P<0.01 and ***P<0.001 vs. control (n is cell number, n = 6). (**B**) Inhibition (%) of peak Kv1.3 currents *vs.* test voltages obtained from panel *A*. The percent inhibition (open squares) are superimposed with the activation curve of Kv1.3 (filled squares) (n = 6). (**C**) The time course of Kv1.3 inhibition by 3 µM DPO-1. Currents were elicited by a 250 ms depolarizing to +40 mV. DPO-1 (3 µM) was applied to the bath while the membrane potential was held at −80 mV. After an interval of 8 min, consecutive 200 ms pulses were applied every 10 s. (**D**) Superimposed current traces recorded in the presence of DPO-1 (pink and red curves) or following washout (in blue) corresponding to panel *C*. The numbers 1 to 17 refers to pulses 1 to 17. (**E**) Kv1.3 current inactivation at +40 mV was fitted with a monoexponential equation, and the time constant has been plotted against DPO-1 concentration (*** P<0.001 vs. control, n = 6). (**F**) Steady-state inactivation curves in the absence (open squares) and presence (filled squares) of DPO-1. Data were obtained from normalized currents at +40 mV, which followed 30 s prepulses to potentials between −80 mV and 0 mV and were fitted with the Boltzman equation (n = 6). Data were expressed as mean±SEM.

We further evaluated the biophysical effects of DPO-1 on Kv1.3 in Jurkat cells. Figure 3*A* shows the current density-voltage relationships, before and after the addition of DPO-1 and after the washout of DPO-1 in Jurkat cells. DPO-1 at 3 µM reduced the Kv1.3 current for the entire voltages range over which this current was activated, and the blocking effect of DPO-1 was completely reversible after washout. Further, the membrane potential was depolarized from −41.07±1.93 mV to −25.99±2.91 mV (P<0.01) and recovered to −41.54±1.60 mV after washout.

To investigate the voltage-dependent block of DPO-1, the percent inhibition of DPO-1 was plotted as a function of membrane potential together with the activation curve. In the presence of DPO-1, inhibition increased steeply between −40 and 0 mV, from 7±7% to 54±5%, corresponding to the voltage range for channel opening. This inhibition reached a steady state between +10 and +60 mV, when the Kv1.3 channels were fully activated (Figure 3*B*). These results suggested that blocking Kv1.3 by DPO-1 treatment is dependent on the voltage of the depolarizing pulse in a manner that indicates an open channel block.

To further determine whether blocking Kv1.3 channels with DPO-1 requires channel opening, we recorded currents in Jurkat cells before and after a pulse-free period of incubation with the compound. Control currents were elicited by repetitive application of depolarizing step pulses at +40 mV. DPO-1 (3 µM) was applied to the bath while the membrane potential was held at −80 mV without any depolarization pulses. After an interval of 8 min to assure a steady-state condition, consecutive 250 ms pulses were applied every 10 s. Figure 3*C and 3D* show that DPO-1 did not affect the amplitude peak current density in the pulse-free conditions, where the amplitude peak Kv1.3 current density at pulse 1 was 62.73 vs. 66.35 pA/pF under control conditions, but significantly accelerated the inactivation of Kv1.3 channels (the time constants of inactivation were 196±2 ms under control and 132±1 ms at pulse 1). The onset of blockade occurred rapidly once the channel was opened with depolarization pulses (the amplitude peak current at pulse 17 was 35.98 vs. 66.35 pA/pF at pulse 1). Moreover, the current recovered quickly upon washout even in pulse-free conditions. These results indicate that DPO-1 preferentially binds to the open state of the Kv1.3 channel.

A double-pulse protocol was used to investigate the effects of DPO-1 on voltage-dependent of steady-state inactivation of Kv1.3 currents in Jurkat cells. DPO-1 at 3 µM caused a negative shift of the steady-state inactivation curve (Figure 3*F*). The half inactivation voltage V_1/2_ were −43±1 mV and −53±1 mV for control and 3 µM DPO-1, respectively (P<0.001 vs. control). The decay phase of the Kv1.3 current at +40 mV was well fitted to a single exponential equation, yielding a time constant of 234±28 ms in control, and 167±17, 95±12, and 66±7 ms in 1, 3, and 10 µM DPO-1, respectively (P<0.001 vs. control for all of the concentrations) (Figure 3*E*). Thus, DPO-1 accelerated the channel inactivation rate in a concentration dependent manner.

### DPO-1 did not block K_Ca_ channel currents in human T cells

**Figure 4:**
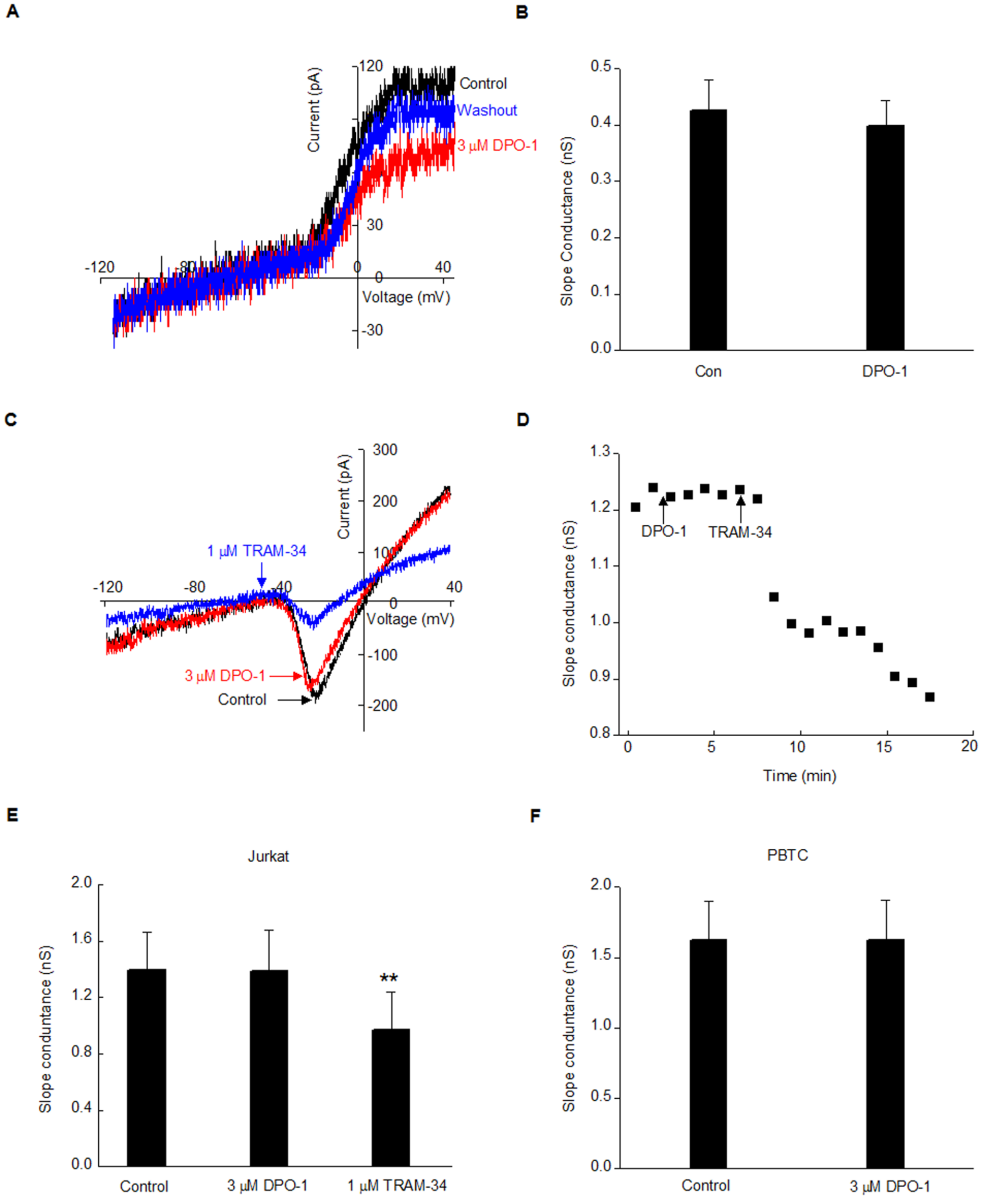
The effect of DPO-1 on K_Ca_ channel currents in human T cells. (**A**) Representative currents-voltage relationships were superimposed before (in black) and after application (in red) with 3 µM DPO-1 in Jurkat cells using normal Ringer's Solution. Currents were elicited by 200 ms voltage-ramps from −120 mV to +40 mV from the holding potential of −40 mV. (**B**) Summarized data from 5 Jurkat cells. (**C**) Representative currents-voltage relationships were superimposed before (in black) and after perfusion with DPO-1 (in red) or TRAM-34 (in blue) in Jurkat cells. Currents were elicited by 200 ms voltage-ramps from −120 mV to +40 mV from the holding potential of −40 mV. (**D**) Time course of the effect of DPO-1 and TRAM-34 on the slope conductance in Jurkat cells. K_Ca_ slope conductance was measured between −120 mV and −40 mV. (**E**) Summarized data from at least 9 Jurkat cell experiments. ** P<0.01 vs. control. (**F**) Summarized data from 7 human peripheral blood T cells. Data were expressed as mean±SEM.

T cells also express the calcium activated potassium channel K_Ca_, which plays a major role in regulating the membrane potential and maintaining Ca^2+^ influx along with the Kv1.3 channel [10], [25]. K_Ca_2.2 is the main K_Ca_ channel involved in the Ca^2+^ signaling of Jurkat cells [26], whereas K_Ca_3.1 is the main K_Ca_ channel in peripheral T cells [25]. Therefore, we investigated whether DPO-1 could also block K_Ca_ currents in both these two kinds of human T cells. Figure 4*A* shows ramp currents in normal Ringer solution before and after application of DPO-1 in Jurkat cells. To avoid any confounding effects of the Kv currents, we measured the K_Ca_ slope conductance between −120 mV and −60 mV and fitted with the results as a linear function. DPO-1 at 3 µM had no apparent effects on the slope factor for K_Ca_ (P = 0.709). To increase the slope conductance for K_Ca_ and make it more accurate to measure, we used the high K^+^ Ringer solution, which was also widely used in many studies [26], [27]–[29] Application of 1 µM TRAM-34, a selective K_Ca_ channel blocker [29], robustly decreased the K_Ca_ slope conductance (Figure 4*A and 4B*). In contrast, DPO-1 at 3 µM had no effects on K_Ca_ in both Jurkat cells and human peripheral blood T cells (Figure 4*C and 4D*), which was also confirmed after the blockade Kv1.3 currents in the presence of MgTX (Figure S1). The slope conductance for Jurkat cells was 1.39±0.07 nS in control group, and 1.39±0.09 nS in the DPO-1 group (P = 0.884). Likewise, slope conductance for human peripheral blood T cells was 1.62±0.28 nS in the control group and 1.62±0.29 nS in cells treated with DPO-1 (P = 1.000).The results for the control T cells were similar to previous reports [28], [30]. Therefore, our results suggest that DPO-1 had no effect on K_Ca_ either in physiological or high K^+^ external solution.

### Kv1.3 channel current density and protein expression was inhibited by pre-treatment with DPO-1 in Jurkat cells

**Figure 5:**
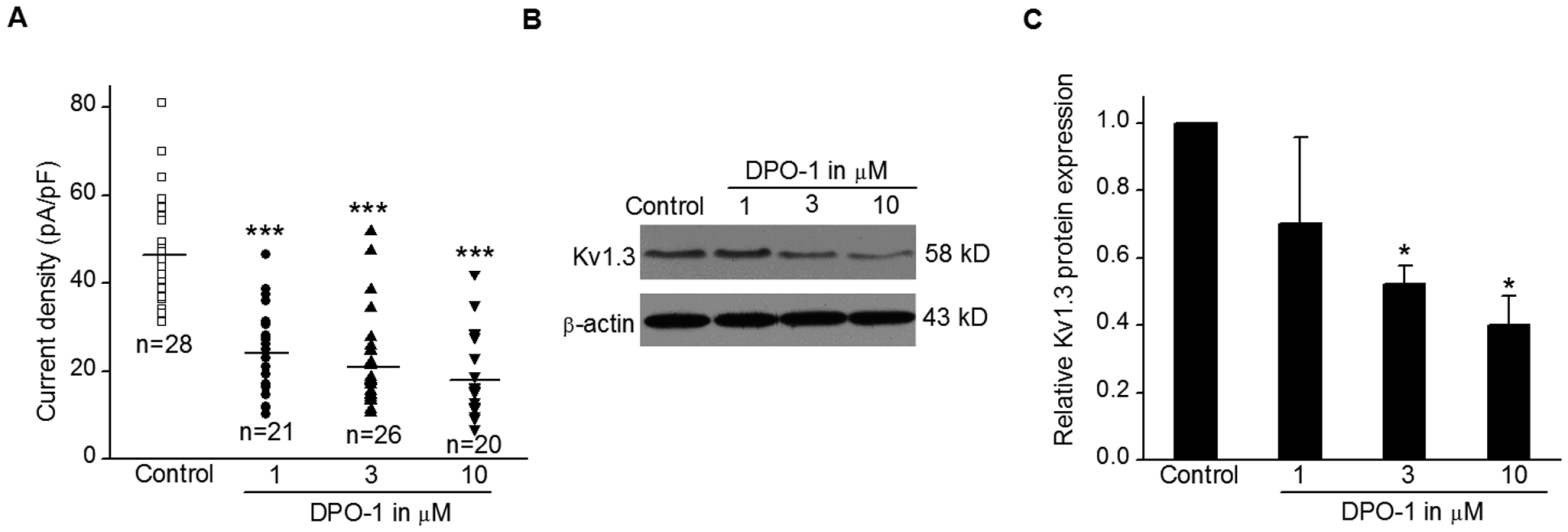
Effects of DPO-1 pre-treatment on Kv1.3 current density and protein expression in Jurkat cells. (**A**) Kv1.3 peak current density at +40 mV under control condition (open squares) and pretreated 24-h with 1 (filled circles), 3 (filled triangles) and 10 (filled inverted triangles) µM DPO-1. n is the cell number, ***P <0.001 vs. control. (**B**) Representative western blot analysis of Kv1.3 protein under control condition and pretreated 24-h with 1, 3, and 10 µM DPO-1. (**C**) Data from 3 experiments replicates were normalized to the amount of β-actin (*P<0.05 vs. control). Data were expressed in mean±SEM.

We further investigated the effects of 24 h DPO-1 pre-treatment on Kv1.3 channel currents and protein expression levels in Jurkat cells. Similar to acute administration, pre-incubation with DPO-1 also blocked the Kv1.3 channel current in a concentration-dependent manner. The current density at +40 mV decreased from 48.52±2.31 pA/pF in control cells to 24.37±2.21, 21.77±2.21 and 18.54±2.10 pA/pF in 1, 3, and 10 µM DPO-1 treated cells, respectively (P<0.001 vs. control for all concentrations) (Figure 5*A*).

Immunoblotting was used to analyze the protein expression of Kv1.3 channel (Figure 5*B* and Figure 5*C*). Pre-treatment with 3 and 10 µM DPO-1 for 24 h decreased Kv1.3 channel protein expression by 47.9% and 60.0%,respectively, (P<0.05 vs. control for both concentrations). However, 30 min pre-treatment with DPO-1 and 24 h pre-treatment with MgTX had no significant effects on Kv1.3 channel protein expression (Figure S2 and S3).

### The Ca^2+^ influx to Ca^2+^-depletion Jurkat cells was blunted by DPO-1

**Figure 6:**
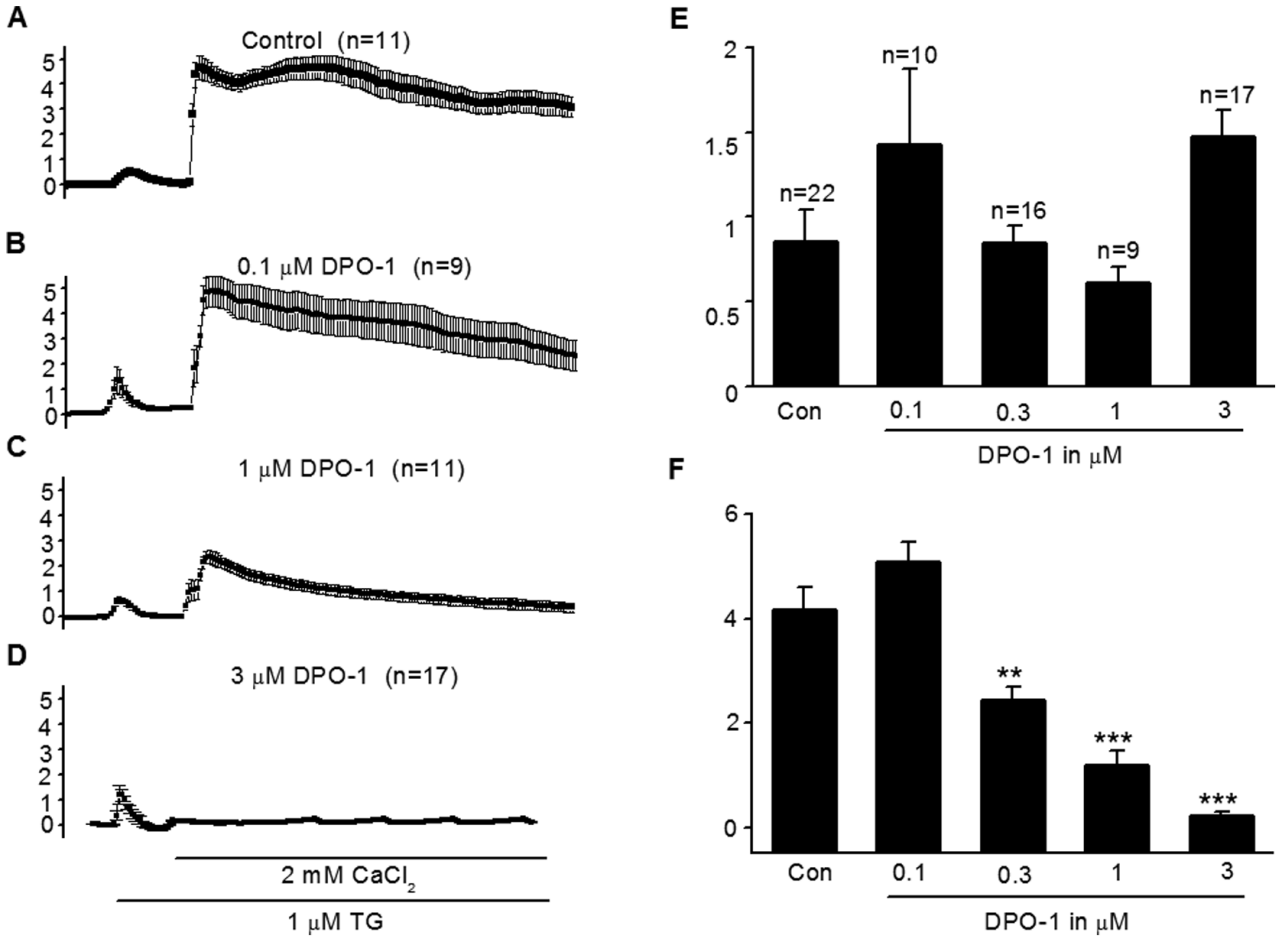
Inhibitory effect of DPO-1 on calcium release and influx in Jurkat cells. Ca^2+^ release was induced by 1 µM thapsagargin (TG) in Ca^2+^ free Ringer solution in Jurkat cells with or without 24 h pre-treatment of DPO-1. After 5 min, normal Ringer solution (2 mM CaCl_2_) was reintroduced, causing a rapid and sustained Ca^2+^ influx. (**A**) Average Ca^2+^ responses as the index △F/F_0_ were shown in control, (**B**) 0.1 µM DPO-1, (**C**) 0.3 µM DPO-1, and (**D**) 3 µM DPO-1. (**E**) Summarized data of peak Ca^2+^ release with TG. (**F**) Summarized data of the peak Ca^2+^ influx with addition of 2 mM Ca^2+^, *P<0.05 and **P<0.01 vs. control. Data were expressed in mean±SEM. n is the cell number.

The Kv1.3 channel controls Ca^2+^ homeostasis in T cells, and Kv1.3 channel inhibition significantly reduces Ca^2+^ influx [10]. To confirm that DPO-1 inhibited Ca^2+^ influx, we measured intracellular Ca^2+^ concentration by laser confocal microscopy. Depletion of Ca^2+^ from the endoplasmic reticulum was induced by 1 µM TG in Ca^2+^-free Ringer's solution. In the absence of extracellular Ca^2+^, TG induced a very small, transient intracellular Ca^2+^ concentration rise in Jurkat T cells. When 2 mM CaCl_2_ was applied to the Ca^2+^-free Ringer's solution, Ca^2+^ flowed into the cell through the Ca^2+^ release-activated Ca^2+^ channel (CRAC) channel to significantly increase intracellular Ca^2+^, as illustrated in Figure 6*A*. Pre-treatment with DPO-1 for 24 h led to a concentration-dependent inhibition of Ca^2+^ influx through the CRAC channel in Ca^2+^-depleted Jurkat cells,but did not alter TG-induced intracellular store Ca^2+^ release (Figure 6). The peak ΔF/F_0_ value representing Ca^2+^ influx decreased from 4.17 in control to 2.4, 1.2, and 0.2, in 0.3, 1 and 3 µM DPO-1, respectively (P<0.01 vs. control for 0.3 and P<0.001 for 1 and 3 µM). Similar results were also found in the pre-treatment of DPO-1 for 30 min (Figure S4).

### DPO-1 inhibited the secretion of IL-2 in activated Jurkat cells

**Figure 7:**
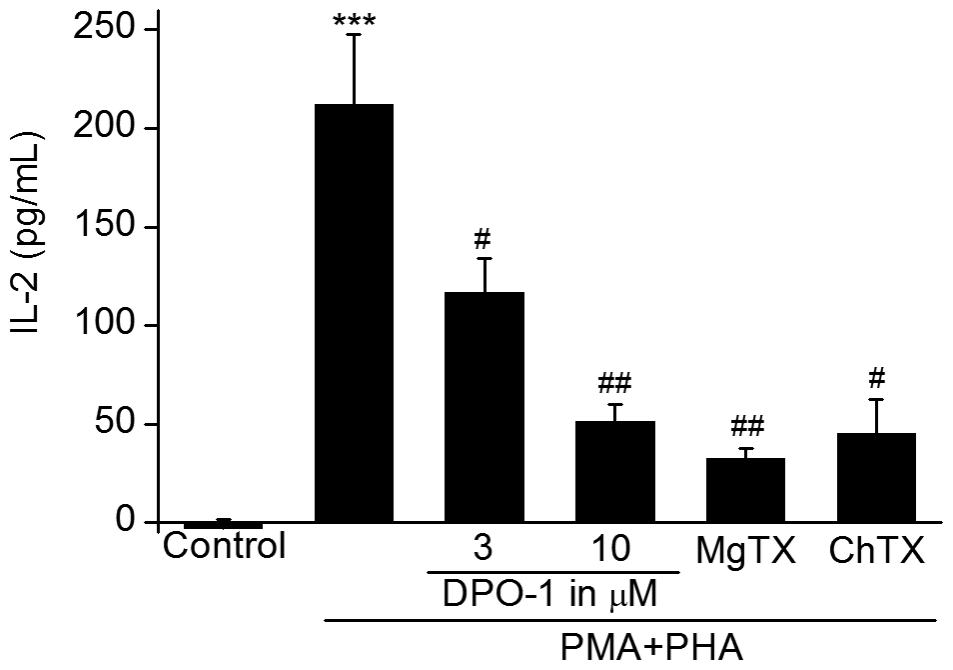
Inhibitory effect of DPO-1 and potassium channel blockers on IL-2 production in activated Jurkat cells. Jurkat cells were activated with PHA (5 µg/mL) and PMA (80 nM) for 24 h. DPO-1 (3 and 10 µM), MgTX (10 nM) and ChTX (100 nM) were added simultaneously. *** P<0.001 vs. control. # P<0.05 and ## P<0.01 vs. activated group. Data were expressed as mean±SEM.

To determine whether Kv1.3 channel and Ca^2+^ influx inhibition by DPO-1 could result in functional immunosuppression, we measured IL-2 secretion in the growth media by ELISA. As shown in Figure 7, IL-2 secretion was increased robustly upon stimulation with PHA+PMA (P<0.001), which served as the positive control. IL-2 levels were significantly reduced in the presence of 3 (P<0.05) and 10 (P<0.01) µM DPO-1 pre-treatment for 24 h. IL-2 secretion was also reduced with 10 nM MgTX and 100 nM ChTX, two known Kv1.3 blockers [31].

## Discussion

This is the first study to investigate the effect of DPO-1, a novel Kv1.5 blocker, on potassium channels in human T cells. Our results demonstrated that DPO-1 blocked the Kv1.3 channel without any significant effect on the K_Ca_ channel. Similar to our previous observation on the Kv1.5 channel, DPO-1 blocked the Kv1.3 channel in a voltage- and concentration-dependent manner, preferentially blocking the open channel state [1]. Pre-treatment with DPO-1 for 24 h significantly reduced Kv1.3 current density and protein expression. Considering the importance of Kv1.3 in triggering Ca^2+^ influx and IL-2 synthesis, we further investigated the effects of DPO-1 on Ca^2+^ influx and IL-2 production. We found that DPO-1 inhibited Ca^2+^ influx as well as IL-2 secretion. Therefore, our results suggested that in addition to inhibiting Kv1.5 channels, DPO-1 has marked inhibitory effects on Kv1.3 currents and its expression, Ca^2+^ influx and IL-2 production.

DPO-1 blocked the Kv1.3 channel in a voltage-dependent manner, and this inhibitory effect increased steeply from voltages of −40 mV to 0 mV. Moreover, DPO-1 did not produce any obvious effect until the channel was first opened by a depolarization pulse, indicating that DPO-1 did not affect the Kv1.3 channel in the closed state. Combined, these effects suggest that DPO-1 interacts with the open state of Kv1.3 channels, which is the same mechanism previously observed for the Kv1.5 channel [1]. Of note, DPO-1 failed to cause a crossover phenomenon of tail current traces, which is in contrast with the properties of other open-channel blockers of the Kv1.3 channel [20], [32]. One possible explanation is that the functional dissociation of DPO-1 from the Kv1.3 channel at repolarization may not interfere with the transition of the channel from an open to a closed state. In addition, our data show that DPO-1 not only accelerated the time course of inactivation in a concentration-dependent manner but also shifted the voltage-dependent steady-state inactivation in the negative direction, indicating that DPO-1 also binds to the inactivated-state. Previous studies have shown that FK-506 [33] and verapamil [34], binding to both the open and inactivation states, caused significant negative shift of the voltage-dependence of steady-state inactivation. Unlike DPO-1, staurosporine [35] and fluoxetine [32], the pure Kv1.3 open channel blockers, had no effects on the voltage-dependence of steady-state inactivation. However, the interactions between DPO-1 and the inactivation state require more sophisticated kinetic analysis.

In addition to the Kv1.3 channel, human T cells express K_Ca_ channels that help set up the membrane potential to drive Ca^2+^ influx necessary for IL-2 synthesis [10], [25]. Our electrophysiological data shows that acute application of 3 µM DPO-1 blocked the Kv1.3 channel, but had little effect on the K_Ca_ channel. Meanwhile, the membrane potential was depolarized from −41.07±1.93 mV to −25.99±2.91 mV. In human T cells, a small fraction of Kv1.3 channel is active at rest to maintain the membrane potential [25], [36]. Therefore, we assumed that DPO-1 could block Kv1.3 channel even in the rest potential range. In addition, membrane depolarization by blocking Kv1.3 has been confirmed to be an effective method to prevent T-cell activation and therefore has applications in many autoimmune conditions [11]. DPO-1 also significantly decreased Ca^2+^ influx and IL-2 secretion in activated Jurkat cells, leading to the attenuation of the immune response. Consistent with other reports, both MgTX and ChTX blocked Kv1.3 to inhibit IL-2 secretion [31]. Furthermore, we also demonstrated that pre-treatment with DPO-1 for 24 h but not 30 min significantly reduced the channel protein expression in T cells. Unlike DPO-1, pre-treatment with MgTX for 24 h had no effect on Kv1.3 protein expression. In addition, pre-treatment with DPO-1 for 24 h had no effects on Kv1.3 mRNA expression (Figure S5). These results suggested thatdirect and acute blockade of Kv1.3 channel by DPO-1 can lead to obvious inhibition of IL-2 secretion. Meanwhile, the inhibitory effect of DPO-1 on Kv1.3 protein expression may strengthen its immunosuppression potential. Overall, our data indicate that DPO-1, by blocking the Kv1.3 currents, down-regulating the Kv1.3 protein expression, and depolarizing membrane potential, blunts Ca^2+^ influx and results in decreased production of IL-2.

Both Kv1.3 and K_Ca_ channels play crucial roles in T cell activation. However, the relative contribution varies in certain T cell subtypes. The activation of T_EM_ cells (the major pathogenic T cells in tissue sites of inflammation [37]) primarily depends on Kv1.3 channels, while K_Ca_ channels have a major role in the activation of naïve and central memory T cells [38]. Importantly, potent and selective Kv1.3 inhibition preferentially prevents T_EM_ function, whereas naïve and central memory T cells (necessary for physiological immune responses) escape inhibition by augmenting K_Ca_ expression [27]. In animal experiments (rats and monkeys), effective dose of DPO-1 as a Kv1.3 blocker showed no apparent cardiac side effects, except for prolonging atrial refractory period [39]. Therefore, the Kv1.3 channel may be considered an attractive target for immunomodulatory therapies [18], [40].

In summary, our study provides the first evidence that Kv1.5 blocker DPO-1 suppresses Kv1.3 channels and exerts immunomodulatory effects by the inhibition of calcium influx and stimulation induced IL-2 production. Our results suggest that DPO-1 may be effective in inflammatory autoimmune diseases and benefits for the development of new drug on immunoregulatory therapy.

## Supporting Information

Figure S1 **Block of K_Ca_ currents with DPO-1 in the presence of MgTX in high K^+^ solution.** First, 1 nM MgTX was applied to the external solution to inhibit Kv1.3 currents (in red), then, 3 µM DPO-1 was added in the presence of MgTX (in blue).
(TIF)

Figure S2 **30 min incubation with DPO-1 had no inhibitory effect on Kv1.3 protein expression.** 1, 3, 10 µM DPO-1 was applied to incubate Jurkat cells for 30 min. Then the Kv1.3 protein expression level was determined by western blot.
(TIF)

Figure S3 **24 h incubation with MgTX had no inhibitory effect on Kv1.3 protein expression.** 10 nM MgTX was applied to incubate Jurkat cells for 24 h. Then the Kv1.3 protein expression level was determined by western blot.
(TIF)

Figure S4 **30 min incubation with DPO-1 inhibited the Ca^2+^ influx in Ca^2+^-depleted Jurkat cells.** Jurkat cells were loaded with 2 µM fluo-3 AM for 30 min, washed twice and resuspended in Ca^2+^ free Ringer's solution. Then the cells were incubated with 0 or 10 µM DPO-1 for 30 min, with thapsgargin in the solution to induce the Ca^2+^ depletion. The multifunctional enzyme mark instrument (PerkinElmer, USA) was used to measure the Ca^2+^ fluorescence intensity. The normal Ringer's solution with 2 mM CaCl_2_ was applied to induce the Ca^2+^ influx. A, Average Ca^2+^ responses as the index △F/F_0_ were shown in control and 10 µM DPO-1; B, Summarized data of the peak Ca2+ influx with addition of 2 mM Ca^2+^, *** P<0.001.
(TIF)

Figure S5 **DPO-1 had no inhibitory effect on Kv1.3 mRNA expression level.** Jurkat cells were incubated with 1, 3, 10 µM DPO-1 for 24 h. Then the relative Kv1.3 mRNA expression level was determined by real-time PCR.
(TIF)

## References

[1] Du YM, Zhang XX, Tu DN, Zhao N, Liu YJ, et al (2010) Molecular determinants of Kv1.5 channel block by diphenyl phosphine oxide-1. J Mol Cell Cardiol 48: 1111–1120. 20184887 10.1016/j.yjmcc.2010.02.010

[2] Lagrutta A, Wang J, Fermini B, Salata JJ (2006) Novel, potent inhibitors of human Kv1.5 K^+^ channels and ultrarapidly activating delayed rectifier potassium current. J Pharmacol Exp Ther 317: 1054–1063. 16522807 10.1124/jpet.106.101162

[3] Regan CP, Wallace AA, Cresswell HK, Atkins CL, Lynch JJ (2006) In vivo cardiac electrophysiologic effects of a novel diphenylphosphine oxide I_Kur_ blocker, (2-Isopropyl-5-methylcyclohexyl) diphenylphosphine oxide, in rat and nonhuman primate. J Pharmacol Exp Ther 316: 727–732. 16243963 10.1124/jpet.105.094839

[4] Stump GL, Wallace AA, Regan CP, Lynch JJ (2005) In vivo antiarrhythmic and cardiac electrophysiologic effects of a novel diphenylphosphine oxide I_Kur_ blocker (2-isopropyl-5-methylcyclohexyl) diphenylphosphine oxide. J Pharmacol Exp Ther 315: 1362–1367. 16157659 10.1124/jpet.105.092197

[5] Pandit SV, Zlochiver S, Filgueiras-Rama D, Mironov S, Yamazaki M, et al (2011) Targeting atrioventricular differences in ion channel properties for terminating acute atrial fibrillation in pigs. Cardiovasc Res 89: 843–851. 21076156 10.1093/cvr/cvq359 PMC3306132

[6] Liu HL, Lin JC (2004) A set of homology models of pore loop domain of six eukaryotic voltage-gated potassium channels Kv1.1-Kv1.6. Proteins 55: 558–567. 15103620 10.1002/prot.20065

[7] Long SB, Campbell EB, Mackinnon R (2005) Crystal structure of a mammalian voltage-dependent Shaker family K^+^ channel. Science 309: 897–903. 16002581 10.1126/science.1116269

[8] Decher N, Kumar P, Gonzalez T, Pirard B, Sanguinetti MC (2006) Binding site of a novel Kv1.5 blocker: a “foot in the door” against atrial fibrillation. Mol Pharmacol 70: 1204–1211. 16835355 10.1124/mol.106.026203

[9] Decher N, Pirard B, Bundis F, Peukert S, Baringhaus KH, et al (2004) Molecular basis for Kv1.5 channel block: conservation of drug binding sites among voltage-gated K^+^ channels. J Biol Chem 279: 394–400. 14578345 10.1074/jbc.M307411200

[10] Lewis RS, Cahalan MD (1995) Potassium and calcium channels in lymphocytes. Annu Rev Immunol 13: 623–653. 7612237 10.1146/annurev.iy.13.040195.003203

[11] Chandy KG, Wulff H, Beeton C, Pennington M, Gutman GA, et al (2004) K^+^ channels as targets for specific immunomodulation. Trends Pharmacol Sci 25: 280–289. 15120495 10.1016/j.tips.2004.03.010 PMC2749963

[12] Varga Z, Hajdu P, Panyi G (2010) Ion channels in T lymphocytes: an update on facts, mechanisms and therapeutic targeting in autoimmune diseases. Immunol Lett 130: 19–25. 20026117 10.1016/j.imlet.2009.12.015

[13] Rangaraju S, Chi V, Pennington MW, Chandy KG (2009) Kv1.3 potassium channels as a therapeutic target in multiple sclerosis. Expert Opin Ther Targets 13: 909–924. 19538097 10.1517/14728220903018957

[14] Fasth AE, Cao D, van Vollenhoven R, Trollmo C, Malmstrom V (2004) CD28nullCD4^+^ T cells–characterization of an effector memory T-cell population in patients with rheumatoid arthritis. Scand J Immunol 60: 199–208. 15238090 10.1111/j.0300-9475.2004.01464.x

[15] Viglietta V, Kent SC, Orban T, Hafler DA (2002) GAD65-reactive T cells are activated in patients with autoimmune type 1a diabetes. J Clin Invest 109: 895–903. 11927616 10.1172/JCI14114 PMC150925

[16] Azam P, Sankaranarayanan A, Homerick D, Griffey S, Wulff H (2007) Targeting effector memory T cells with the small molecule Kv1.3 blocker PAP-1 suppresses allergic contact dermatitis. J Invest Dermatol 127: 1419–1429. 17273162 10.1038/sj.jid.5700717 PMC1929164

[17] Beeton C, Wulff H, Barbaria J, Clot-Faybesse O, Pennington M, et al (2001) Selective blockade of T lymphocyte K^+^ channels ameliorates experimental autoimmune encephalomyelitis, a model for multiple sclerosis. Proc Natl Acad Sci USA 98: 13942–13947. 11717451 10.1073/pnas.241497298 PMC61146

[18] Beeton C, Wulff H, Standifer NE, Azam P, Mullen KM, et al (2006) Kv1.3 channels are a therapeutic target for T cell-mediated autoimmune diseases. Proc Natl Acad Sci USA 103: 17414–17419. 17088564 10.1073/pnas.0605136103 PMC1859943

[19] Wang X, Liao Y, Zou A, Li L, Tu D (2007) Blockade action of ketanserin and increasing effect of potassium ion on Kv1.3 channels expressed in Xenopus oocytes. Pharmacol Res 56: 148–154. 17582781 10.1016/j.phrs.2007.05.002

[20] Ahn HS, Kim SE, Jang HJ, Kim MJ, Rhie DJ, et al (2007) Open channel block of Kv1.3 by rosiglitazone and troglitazone: Kv1.3 as the pharmacological target for rosiglitazone. Naunyn Schmiedebergs Arch Pharmacol 374: 305–309. 17119927 10.1007/s00210-006-0118-6

[21] Pang B, Zheng H, Shin DH, Jung KC, Ko JH, et al (2010) TNF-alpha inhibits the CD3-mediated upregulation of voltage-gated K^+^ channel (Kv1.3) in human T cells. Biochem Biophys Res Commun 391: 909–914. 19951698 10.1016/j.bbrc.2009.11.162

[22] DeCoursey TE, Chandy KG, Gupta S, Cahalan MD (1984) Voltage-gated K^+^ channels in human T lymphocytes: a role in mitogenesis? Nature 307: 465–468. 6320007 10.1038/307465a0

[23] Matteson DR, Deutsch C (1984) K channels in T lymphocytes: a patch clamp study using monoclonal antibody adhesion. Nature 307: 468–471. 6320008 10.1038/307468a0

[24] Garcia-Calvo M, Leonard RJ, Novick J, Stevens SP, Schmalhofer W, et al (1993) Purification, characterization, and biosynthesis of margatoxin, a component of Centruroides margaritatus venom that selectively inhibits voltage-dependent potassium channels. J Biol Chem 268: 18866–18874. 8360176

[25] Cahalan MD, Chandy KG (2009) The functional network of ion channels in T lymphocytes. Immunol Rev 231: 59–87. 19754890 10.1111/j.1600-065X.2009.00816.x PMC3133616

[26] Grissmer S, Lewis RS, Cahalan MD (1992) Ca^2+^-activated K^+^ channels in human leukemic T cells. J Gen Physiol 99: 63–84. 1371308 10.1085/jgp.99.1.63 PMC2216598

[27] Ghanshani S, Wulff H, Miller MJ, Rohm H, Neben A, et al (2000) Up-regulation of the IKCa1 potassium channel during T-cell activation. Molecular mechanism and functional consequences. J Biol Chem 275: 37137–37149. 10961988 10.1074/jbc.M003941200

[28] Fanger CM, Rauer H, Neben AL, Miller MJ, Rauer H, et al (2001) Calcium-activated potassium channels sustain calcium signaling in T lymphocytes. Selective blockers and manipulated channel expression levels. J Biol Chem 276: 12249–12256. 11278890 10.1074/jbc.M011342200

[29] Wulff H, Miller MJ, Hansel W, Grissmer S, Cahalan MD, et al (2000) Design of a potent and selective inhibitor of the intermediate-conductance Ca^2+^-activated K^+^ channel, IKCa1: a potential immunosuppressant. Proc Natl Acad Sci USA 97: 8151–8156. 10884437 10.1073/pnas.97.14.8151 PMC16685

[30] Robbins JR, Lee SM, Filipovich AH, Szigligeti P, Neumeier L, et al (2005) Hypoxia modulates early events in T cell receptor-mediated activation in human T lymphocytes via Kv1.3 channels. J Physiol 564: 131–143. 15677684 10.1113/jphysiol.2004.081893 PMC1456048

[31] Villalonga N, David M, Bielanska J, Gonzalez T, Parra D, et al (2010) Immunomodulatory effects of diclofenac in leukocytes through the targeting of Kv1.3 voltage-dependent potassium channels. Biochem Pharmacol 80: 858–866. 20488163 10.1016/j.bcp.2010.05.012

[32] Choi JS, Hahn SJ, Rhie DJ, Yoon SH, Jo YH, et al (1999) Mechanism of fluoxetine block of cloned voltage-activated potassium channel Kv1.3. J Pharmacol Exp Ther 291: 1–6. 10490879

[33] Ahn HS, Kim SE, Choi BH, Choi JS, Kim MJ, et al (2007) Calcineurin-independent inhibition of KV1.3 by FK-506 (tacrolimus): a novel pharmacological property. Am J Physiol Cell Physiol 292: C1714–C1722. 17166943 10.1152/ajpcell.00258.2006

[34] Robe RJ, Grissmer S (2000) Block of the lymphocyte K^+^ channel mKv1.3 by the phenylalkylamine verapamil: kinetic aspects of block and disruption of accumulation of block by a single point mutation. Br J Pharmacol 131: 1275–1284. 11090098 10.1038/sj.bjp.0703723 PMC1572478

[35] Choi JS, Hahn SJ, Rhie DJ, Jo YH, Kim MS (1999) Staurosporine directly blocks Kv1.3 channels expressed in Chinese hamster ovary cells. Naunyn Schmiedebergs Arch Pharmacol 359: 256–261. 10344523 10.1007/pl00005350

[36] Verheugen JA, Vijverberg HP, Oortgiesen M, Cahalan MD (1995) Voltage-gated and Ca^2+^-activated K^+^ channels in intact human T lymphocytes. Noninvasive measurements of membrane currents, membrane potential, and intracellular calcium. J Gen Physiol 105: 765–794. 7561743 10.1085/jgp.105.6.765 PMC2216960

[37] Rus H, Pardo CA, Hu L, Darrah E, Cudrici C, et al (2005) The voltage-gated potassium channel Kv1.3 is highly expressed on inflammatory infiltrates in multiple sclerosis brain. Proc Natl Acad Sci USA 102: 11094–11099. 16043714 10.1073/pnas.0501770102 PMC1182417

[38] Wulff H, Calabresi PA, Allie R, Yun S, Pennington M, et al (2003) The voltage-gated Kv1.3 K^+^ channel in effector memory T cells as new target for MS. J Clin Invest 111: 1703–1713. 12782673 10.1172/JCI16921 PMC156104

[39] Regan CP, Stump GL, Wallace AA, Anderson KD, McIntyre CJ, et al (2007) In vivo cardiac electrophysiologic and antiarrhythmic effects of an isoquinoline IKur blocker, ISQ-1, in rat, dog, and nonhuman primate. J Cardiovasc Pharmacol 49: 236–245. 17438409 10.1097/FJC.0b013e3180325b2a

[40] Panyi G, Possani LD, Rodriguez DLVR, Gaspar R, Varga Z (2006) K^+^ channel blockers: novel tools to inhibit T cell activation leading to specific immunosuppression. Curr Pharm Des 12: 2199–2220. 16787250 10.2174/138161206777585120

