# Supplementary figures and images for: Potent Suppression of Kv1.3 Potassium Channel and IL-2 Secretion by Diphenyl Phosphine Oxide-1 in Human T Cells
Source: PLoS One. 2013 May 22;8(5):e64629. doi: 10.1371/journal.pone.0064629 (PMC3661503; doi:10.1371/journal.pone.0064629)

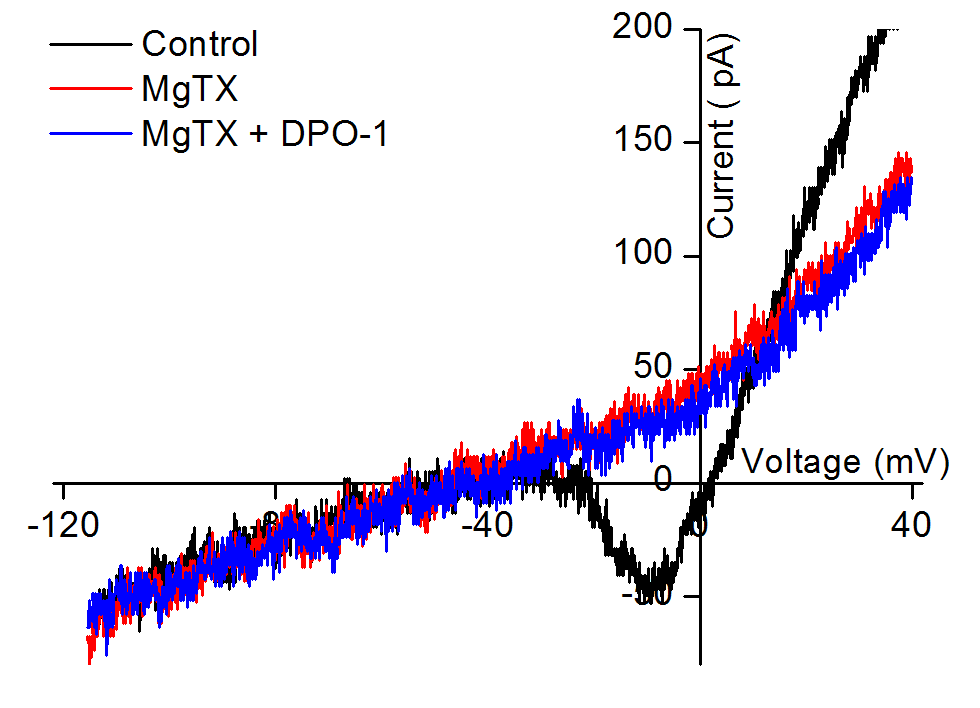

Supplement: Figure S1 — Block of KCa currents with DPO-1 in the presence of MgTX in high K+ solution. First, 1 nM MgTX was applied to the external solution to inhibit Kv1.3 currents (in red), then, 3 µM DPO-1 was added in the presence of MgTX (in blue). (TIF) [file pone.0064629.s001.tif]

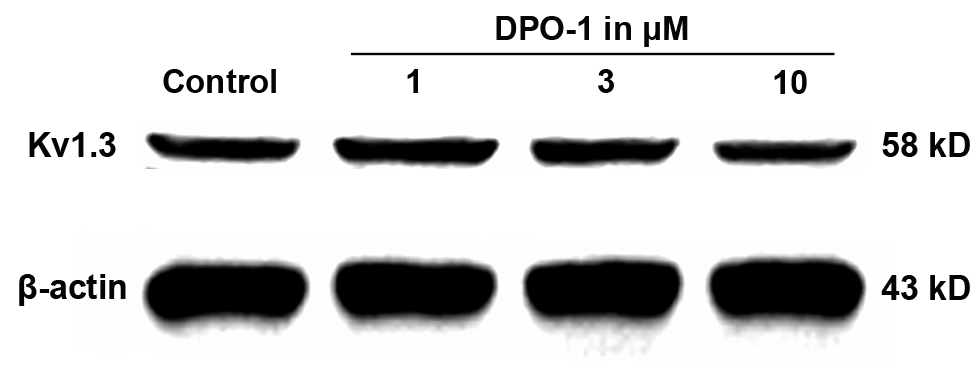

Supplement: Figure S2 — 30 min incubation with DPO-1 had no inhibitory effect on Kv1.3 protein expression. 1, 3, 10 µM DPO-1 was applied to incubate Jurkat cells for 30 min. Then the Kv1.3 protein expression level was determined by western blot. (TIF) [file pone.0064629.s002.tif]

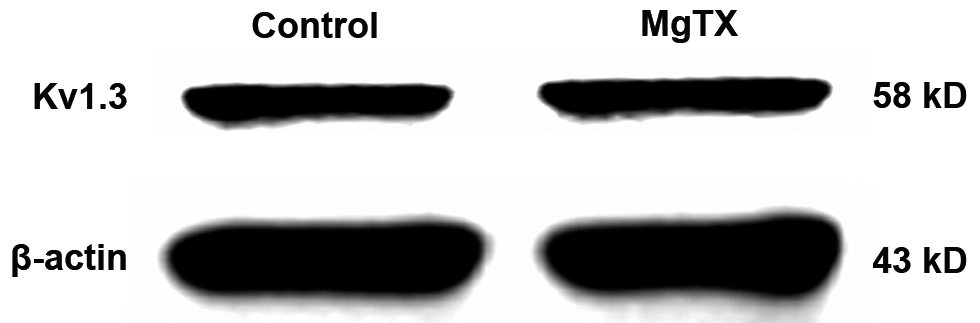

Supplement: Figure S3 — 24 h incubation with MgTX had no inhibitory effect on Kv1.3 protein expression. 10 nM MgTX was applied to incubate Jurkat cells for 24 h. Then the Kv1.3 protein expression level was determined by western blot. (TIF) [file pone.0064629.s003.tif]

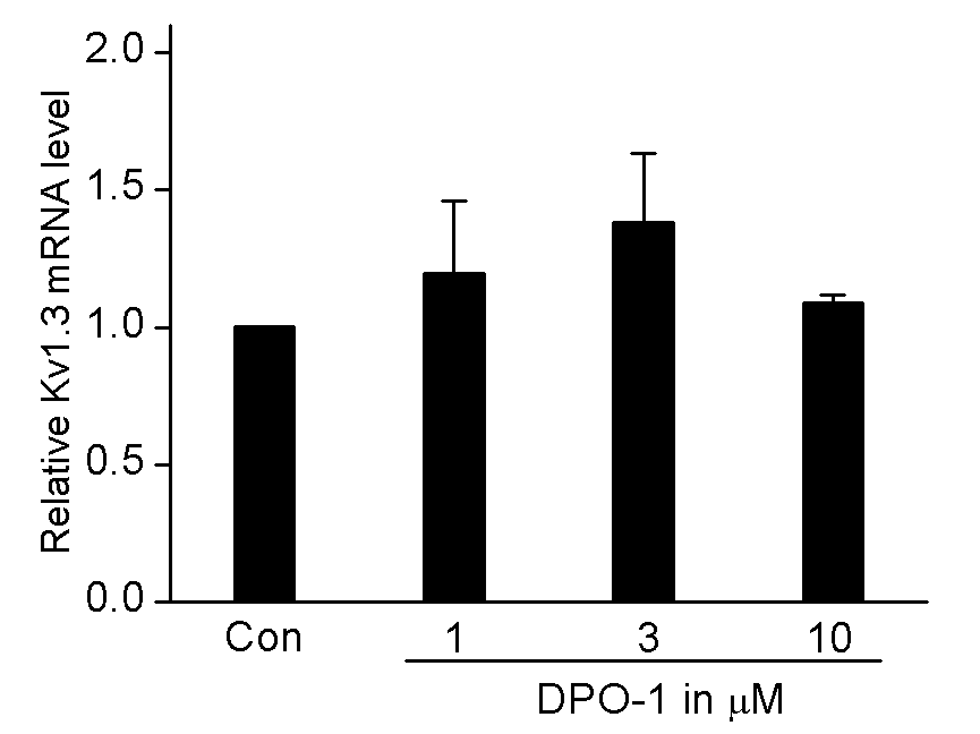

Supplement: Figure S5 — DPO-1 had no inhibitory effect on Kv1.3 mRNA expression level. Jurkat cells were incubated with 1, 3, 10 µM DPO-1 for 24 h. Then the relative Kv1.3 mRNA expression level was determined by real-time PCR. (TIF) [file pone.0064629.s005.tif]
